# Give Us a Rest

**Published:** 1883-10

**Authors:** George E. Blackham


### GIVE US A REST.

GEORGE E. BLACKHAM, M. D.

That is slangy, perhaps, but it voices so accurately and completely the cry of my wearied and exasperated soul that I am fain to pardon its slanginess and to adopt it as my battle-cry. For months past I have not been able to pick up a magazine or other literary periodical without finding myself confronted with one or more articles on Thomas Carlyle, Jane Welsh Carlyle, Carlyle Memoirs, Carlyle's Writings, Carlyle and Emerson, Carlyle, Carlyle, Carlyle, Car-ar-ar-ar-ly-y-le—here, there and everywhere, till I am as sick of the sound as I ever was of "Dear Little Buttercup, Sweet Little Buttercup," "What Never?" and the other delightful but dreadfully overworked extracts from "Pinafore."

Who and what was this idol before whom so large a portion of the intellectual world has been kow-tow-ing and prostrating itself with an elaboration of self-abasement that might be copied from the manner of a newly-made mandarin upon his first appearance at the court of his Celestial Highness, the half brother of the Sun and Moon, etc.? Well, so far as I can gather from the writings of his worshippers, he was an objectionable Scotchman, with bad manners, bad temper and bad digestion—a man so wrapped up in a morbid egotism as to believe, or to affect to believe, that he had a special mission from the Almighty, and was therefore absolved from the little courtesies and kindnesses that render human intercourse pleasant, and even endurable.

A constant prater about honesty and reform, he was himself a seeker for an office for which he was not qualified, and he abused his friend Jeffrey in a way that would have filled the heart of a Billingsgate fish-woman with envy, when Jeffrey refused to use his influence to get him (Carlyle) appointed to the position of astronomer in an observatory!

That Carlyle was disagreeable, obstinate, hot-tempered and selfish, his admirers all admit; that his literary style was uneven, and was most effective when most abusive, no one appears to deny; that he fell so in love with the German language that he attempted to inoculate our good English tongue with some of its clumsiest idioms, his writings bear witness. A pessimist, believing the worst of all mankind, save only his own sweet self; a sham, modern Jeremiah, going about prophesying all manner of discomfort for everybody, and seeing, too, so far as his own personal influence could extend, that his prophesies were fulfilled.

Married to a woman of high intellect and a warm, loving heart, he let her toil and slave for him like a bond servant, and die hungry for some evidence that he reciprocated, or at least appreciated, her unexplainable affection for him. Neglecting her as he did, giving her cause for jealousy, as he seems to have done, he made her life wretched, and after her death he breaks out into unmanly hysterics, not for her, but for "*his own* loss."

As a writer, he appears to have been a chronic fault-finder and grumbler. Much of his writing is so obscure and involved that his meaning is disputed about even among the inner circle of his disciples. Usually, rising to eloquence only in denunciation, his statue, if erected at all, should be placed in the fish market at Billingsgate as the patron saint and exemplar of the place.

Thus far his history has been written only by his friends and disciples, and this appears to be a fair summing up of what they have said of him. What the record might be if written by his enemies, it is hard to guess. Certain it is, that the more the details of his life are brought out, the more disagreeable the picture becomes; and our literary editors should see that, in mercy to him and their long-suffering readers, it were best to say:

"Nothing in his life became him like the leaving of it"

And, having thus recorded his chief service to his kind, give him, and us, a rest, and fill their pages with matter more agreeable or more profitable.

